# The utility of extended differential parameters as a biomarker of bacteremia at a tertiary academic hospital in persons with and without HIV infection in South Africa

**DOI:** 10.1371/journal.pone.0262938

**Published:** 2022-02-17

**Authors:** Lauren Lemkus, Denise Lawrie, Jenifer Vaughan

**Affiliations:** 1 Department of Molecular Medicine and Haematology, University of the Witwatersrand, Johannesburg, South Africa; 2 National Health Laboratory Services, Johannesburg, South Africa; Tulane National Primate Research Center, UNITED STATES

## Abstract

**Introduction:**

Extended differential parameters (EDPs) are generated with the automated differential count by Sysmex XN-series automated hematology analysers, and include the immature granulocyte count (IG%), the neutrophil fluorescent light intensity (NE-SFL) and the neutrophil fluorescent light distribution width (NE-WY). These have been proposed as early biomarkers of bacteremia. This study aimed to evaluate the NE-SFL, NE-WY and IG% in comparison to neutrophil CD64 (nCD64) expression (as a high quality sepsis biomarker) among patients with suspected bacterial sepsis at the Chris Hani Baragwanath Academic Hospital in Johannesburg, South Africa.

**Methods:**

A daily search of the laboratory information system identified samples submitted for a blood culture (BC) and a concurrent full blood count (FBC). Automated differential counts using a Sysmex XN-9000 haematology analyser and neutrophil CD64 expression by flow cytometry were assessed on the residual FBC samples.

**Results:**

A total of 151 samples were collected, of which 83 were excluded due to equivocal results with regards to the presence of bacterial infection. The remaining 68 samples included 23 with bacteremia, 28 with evidence of non-bacteremic bacterial infection, 13 with no evidence of bacterial infection and 4 with Tuberculosis. HIV status was documented in 90 of the patients, with a seropositivity rate of 57.8%. The EDPs were all significantly higher among patients with bacteremia as compared to those without bacterial infection, but on ROC curve analyses, only the NE-SFL showed good performance (AUC>0.8) for discriminating cases with bacteremia from those without bacterial infection at a cut-off value of 49.75. In comparison to the nCD64, the NE-SFL showed moderate agreement (kappa = 0.5). On stratification of the ROC analysis by HIV status, the NE-SFL showed superior performance among persons with HIV infection (AUC = 1), while the automated IG% showed better performance among the patients without HIV infection (AUC = 0.9).

**Conclusion:**

In this study, EDPs showed differential performance as biomarkers for bacteremia according to HIV-status in the South African setting, with the most promising results seen with the NE-SFL and IG% parameters among people with and without HIV infection, respectively. Further assessment of these parameters without pre-selection of patients likely to have infection is required to further determine their clinical utility, particularly among patients with underlying inflammatory conditions or malignancy.

## Introduction

Bacteremia is defined as the presence of live bacteria circulating within the bloodstream [1]. It develops when bacteria succeed in evading the immune system of the host or when the immune response is insufficient to halt bacterial spread due to intrinsic or acquired immune defects [2]. Bacteremia can result in severe organ dysfunction due to a dysregulated immune response to the infection, which is then termed bacterial sepsis. If bacteremia and sepsis are not identified early and rapidly managed, they can result in septic shock, multiple organ failure and death [3]. Sepsis is estimated to affect >48 million people globally every year, being potentially lethal in >10 million cases [4]. It is best diagnosed by means of a positive blood culture (BC), which is considered to be the “gold standard” test for detecting bacteremia. However, a significant limitation of the BC is the lengthy incubation period [5], and additional laboratory tests (septic biomarkers) are therefore often used to screen for infection earlier in its course. Septic biomarkers are laboratory measured analytes or parameters which can be used to predict the likelihood of sepsis being present. No septic biomarkers are 100% sensitive or specific, but rather serve as a more rapid screening test used to determine whether antibiotic treatment should be commenced while the culture results are awaited. Commonly used septic biomarkers include the full blood count (FBC) and differential white cell count (to detect leucocytosis and neutrophilia), peripheral blood smear review (to detect morphological features of infection such as toxic granulation and band cells), C-reactive protein (CRP) levels and the soluble serum marker procalcitonin (Pct) [6]. However, these tests have weaknesses; many have poor specificity, as they may be elevated in both bacterial and non-bacterial infection as well as in non-infectious inflammatory disease processes (such as auto-immune conditions and cancer), and some (particularly the Pct) are costly [6, 7]. A less commonly used sepsis biomarker is assessment by flow cytometry of the expression of CD64 on the surface of neutrophils (nCD64) in response to acute systemic inflammatory activation [6], which out-performs both the CRP and Pct in sepsis detection [8]. CD64 is a high-affinity immunoglobulin Fc gamma receptor which is upregulated on activated neutrophils, with expression being low on resting neutrophils. Some studies have augmented nCD64 testing by assessing the concurrent decrease in monocyte HLA-DR (mHLA-DR) expression, which occurs due to a compensatory anti-inflammatory effect in sepsis [9, 10]. Unfortunately, like the Pct, these flow cytometry-based tests are expensive, and also require specialised laboratory facilities.

Other newer potential infectious biomarkers include those generated on white cell analysis by automated hematology analysers, namely extended differential parameters (EDPs). These reflect differences detected in size or fluorescence of the various types of white cells during white cell counting and differentiation. These hold appeal as biomarkers of sepsis in resource constrained environments, as they are routinely performed with the automated differential count and therefore incur no additional costs. In general, the EDPs which have been shown to have some value reflect either neutrophil activation or granulocytic left shift, both of which are important changes which occur in the acute immune response to bacterial infection. Sysmex haematology analysers (Sysmex, Kobe, Japan) are widely used in Africa, and generate a number of EDPs. These include the neutrophil fluorescent light intensity (NE-SFL) and the neutrophil fluorescent light distribution width (NE-WY) (both of which are postulated to reflect neutrophil activation [11]), and the automated immature granulocyte (IG) count (which enumerates granulocyte precursors (promyelocytes, myelocytes and metamyelocytes) [12, 13], and consequently reflects granulocytic left shift). All three of these parameters have previously been shown to have some utility as sepsis biomarkers in the developed world [5, 11, 13–19], but have not to date been evaluated in Africa to our knowledge. However, a South African study showed that the automated IG count was inaccurate in a substantial proportion of patients, particularly in those with underlying human immunodeficiency virus (HIV) infection [20]. As South Africa (SA) has a high burden of HIV-infection (with an estimated 7.97 million people (~13.5% of the population) living with HIV [21]), the utility of the EDPs as sepsis biomarkers in the South African context requires verification.

Also of interest in SA is the performance of the EDPs in differentiating bacterial sepsis from Tuberculosis (TB), as these two entities can mimic one another clinically. TB is highly prevalent in SA, with an incidence of 615 cases per 100 000 in 2019 [22]. Like bacterial sepsis, TB is typified by a pronounced acute phase response, and is associated with neutrophil activation [23]. However, in contrast to bacterial sepsis, TB is associated with a more prominent monocyte response, with both the ratio of monocytes to lymphocytes as well as expression of monocyte CD64 being increased in individuals with active TB [24]. Assessing for evidence of monocyte activation may thus be of additional value among patients with suspected bacterial sepsis in settings with a high prevalence of TB. The Sysmex EDP assessing monocyte fluorescence (MO-Y) may be of value in this regard, as its level is proportional to the amount of DNA and RNA in the cell, which could reflect the degree of monocyte activation. To date, none of the EDPs have been assessed among patients with TB to our knowledge.

In this study, we aimed to assess the usefulness of EDPs as biomarkers of bacteremia in the South African setting as compared to nCD64 expression (as a high quality sepsis biomarker). As secondary objectives, we also aimed to evaluate whether the NE-SFL and MO-Y levels truly do reflect neutrophil and monocyte activation respectively, and whether the MO-Y may be of value in discriminating bacterial sepsis from TB.

## Methods

### Sample collection and processing

The study was performed at the Chris Hani Baragwanath Academic Hospital (CHBAH), which is a large academic state-sector hospital in Johannesburg, SA. Samples submitted to the National Health Laboratory Service (NHLS) laboratory for a BC in standard aerobic bottles from patients admitted to the CHBAH were identified by a daily search of the NHLS laboratory information system (LIS). A search for an FBC sample submitted within 12 hours of the BC request was made, and an automated differential count performed using a Sysmex XN-9000 hematology analyser where a differential count had not been performed up-front. Samples were not included if they had an insufficient volume (<1 ml) following routine testing or if the analyser failed to perform a differential count. Two slides were then made and stained with May-Grünwald-Giemsa and a manual differential count performed by two competent morphologists on 200 cells each if the automated IG count was >1.0%. This was done in order to determine a manual immature granulocyte count, which was calculated as the average of the sum of promyelocytes, myelocytes and metamyelocytes counted manually by each morphologist. The two morphologists’ results were compared by means of a Rümke table, and were concordant in all but two cases. For these two cases, a manual differential count was performed by a third competent morphologist and the more concordant results utilised. The automated and mean manual results were compared by means of a Rümke table, and were classified as grossly discordant if the analyser IG count fell ≥2% beyond the range specified in the Rümke table.

Neutrophil CD64 expression was measured on the same sample by flow cytometry using an adaptation of a previously described protocol [9] on a Beckman Coulter Navios flow cytometer (Beckman Coulter, Inc, Brea, California, USA). The mean fluorescent intensity (MFI) for CD64 and HLA-DR was recorded for neutrophils, lymphocytes and monocytes with a latex bead included in each sample to confirm fluorescence stability between analyses. Lymphocyte CD64 (lCD64) was used as an internal negative control, as lymphocytes do not express CD64 under any circumstances. The ratios of nCD64 and monocyte CD64 (mCD64) to lCD64 were calculated to compensate for minor variances of antigen MFI between samples. The ratio of nCD64 to mHLA-DR was also recorded. A more detailed description of the flow cytometry methods can be viewed in the S1 Appendix. Analysis was performed within 36 hours after blood sampling to ensure viability of cellular components. Neutrophil CD64 expression has been previously shown to be stable for up to 72 hours [25]. Stability of the EDP parameters was assessed on 10 randomly selected samples measured at 6 hour intervals from 0 to 24 hours, at 12 hour intervals up to 48 hours and then at 24 hour intervals to 72 hours. Five of the samples were kept at room temperature, and 5 were refrigerated at 2–8°C after the index analysis. This confirmed all the parameters to be stable (with a median change in all results of <10%) for at least 24 hours when stored at room temperature, and at least 48 hours when refrigerated (S1 Table).

Pertinent information was recorded from the LIS (including HIV status, CRP and Pct levels, relevant clinical details (where available) as well as other culture results (including TB culture and Gene-Xpert MTB/RIF assay results (Cepheid, Sunnyvale, California)).

### Sample classification

Samples were divided into 3 groups according to the BC and other laboratory results, namely a bacteremic group (with a positive BC), a non-bacteremic bacterial infection group (with a negative BC but with other clinical or laboratory evidence of bacterial infection (such as positive culture from another site), and a bacterial infection-negative group (with no laboratory or clinical evidence of bacterial infection). Only samples that grew a pathogenic organism were included in the bacteremic group. Cases with negative or contaminated cultures with an elevated CRP or Pct were regarded as being indeterminate as regards the presence of bacterial infection, and were excluded from the EDP and nCD64 performance analysis (as elevation of these biomarkers is not specific for infection) [6, 26].

### Sample size calculation

The target sample size was set at 150 specimens for this study, as this is a comparable sample size to that used in several prior studies evaluating various parameters as sepsis biomarkers [5, 11, 27, 28]. Assuming a positive blood culture rate of 30%, this sample size was anticipated to allow detection of a minimum test sensitivity of 80% (+/-10%) at a 90% confidence level using the following formula: N = 4(1.65)^2^P(1-P)/(0.2)^2^.

### Diagnostic cut-off selection

While sensitivity is often prioritized over test specificity for diagnostic biomarkers used for screening purposes (with the “gold standard” tests reserved to more definitively prove or disprove the presence of disease in screen-positive cases), this is less applicable in the setting of bacterial sepsis, which requires prompt clinical action. Septic biomarkers are therefore often performed concurrently with BCs (the “gold standard” test), and are used to determine whether antibiotic treatment should be commenced while the culture results are awaited. Prioritizing high test sensitivity at the expense of reasonable test specificity in this setting can therefore result in inappropriate antibiotic use. Consequently, cut-off values with the best balance between test sensitivity and specificity for identifying either bacterial infection or bacteremia (as evidenced by a high likelihood ratio (LR) with both high sensitivity and high specificity) were selected for the EDPs and the flow cytometry markers shown to be significantly different in patients with bacteremia (the nCD64:lCD64, mCD64:lCD64 and nCD64:mHLA-DR) in this study. Only samples with unequivocal results (bacteremic, non-bacteremic bacterial infection and bacterial infection negative cases) were included to determine the cut-off values. The diagnostic test performance was assessed by means of the area under the curve (AUC) on receiver operating characteristics (ROC) analysis. Test sensitivity, specificity, as well as positive and negative predictive values are reported.

### Statistical analysis

Continuous data are presented as the median (interquartile range (IQR)) and categorical data as frequencies and percentages.

The agreement between the EDPs and the presence of an increased nCD64:mHLA-DR ratio (as the biomarker which showed the best diagnostic performance) was assessed by Cohen’s kappa co-efficient in all samples (including those with equivocal results regarding the presence of bacterial infection) using the optimal cut-off values for detection of bacteremia (as determined on the ROC analysis). Values of <0.2 were considered to show no to slight agreement, 0.21–0.4 fair agreement, 0.41–0.6 moderate agreement and values of 0.61–0.8 substantial agreement. The Mann-Whitney U-test was used to compare continuous variables of interest. The correlation between the NE-SFL and nCD64:lCD64 and the MO-Y and mCD64:lCD64 (as evidence of neutrophil and monocyte activation, respectively) was assessed by linear regression analysis. Statistical significance was accepted at a p-value <0.05.

Statistical analysis was performed using Prism software, version 5 (GraphPad Software, San Diego, California, United States).

### Ethics

The study was approved by the Human Research Ethics Committee of the University of the Witwatersrand (M-190524 and M-1911201). Data was not anonymous during data collection, but was fully anonymized when recorded. The need for informed consent was waived by the ethics committee.

## Results

### Study population

A total of 151 samples were collected, with a median patient age of 36 years and a male:female ratio of ~0.5:1. The patients were admitted to a variety of wards, including medical (46%), surgical (10%), paediatric (11%), intensive care/high care (13%), maternity/gynaecology (13%), burns (2%), psychiatry (<1%) and oncology (5%) units. The HIV status was documented in 90 of the included samples, with a seropositivity rate of 57.8%. Unequivocal results were present in 68 samples, including 23 in the bacteremic group, 28 in the non-bacteremic bacterial infection group and 13 in the bacterial infection negative group. In addition, 4 patients were proven to have TB, all of whom were persons with HIV infection. Among the patients with HIV, there was no significant difference in the CD4 counts (p = 0.74) or Log HIV viral loads (p = 0.29) between patients with bacteremic infection as compared to those with non-bacteremic bacterial infection. Unfortunately, there were too few patients with HIV infection in the bacterial negative group for CD4 and viral load analysis in this subgroup. Relevant demographic information for the unequivocal cases is shown in Table 1.

**Table 1:** Biometrics and EDP data of all samples with unequivocal results.

Sample group	All bacteremic infection (BC positive)	All non-bacteremic bacterial infection (BC negative, excluding TB)	Proven TB	All bacterial infection negative
**Number of samples**	23	28	4	13
**Male: Female**	0.29:1	0.5:1	1:1	0.7:1
**Median Age [IQR] (years)**	32 (25.5–51.5)	29 (8–54)	36 (29–43)	17 (0.5–26.3)
**HIV positive samples**	8/14 (57.1)	9/16 (56.3)	4/4 (100)	2/7 (28.6)
**(pos/all tested)%**				
**nCD64:lCD64**				
Median	13.0	5.26	20.15	1.6
IQR	9.3–37.	2.6–10.3	13.38–98.75	1.00–4.2
p-value*	0.0003	0.0059	0.005	Not applicable
**mCD64:lCD64**				
Median	41.2	49.6	66.81	36.7
IQR	22.6–78.7	27.8–63.3	30.05–131.2	29.2–47.8
p-value*	0.46	0.28	0.16	Not applicable
**nCD64:mHLA-DR**				
Median	1.12	0.52	3.9	0.17
IQR	0.70–3.10	0.18–1.02	1.76–5.75	0.03–0.30
p-value*	< 0.0001	0.005	0.004	Not applicable
**NE-WY**				
Median	840.0	765	827.5	731.0
IQR	769.0–1002.0	662–900	733.3–865.5	655.5–819.5
p-value*	0.0057	0.32	0.16	Not applicable
**NE-SFL**				
Median	57.0	50.4	54.2	46.2
IQR	54.6–63.9	46.7–53.2	48.7–60.6	42.3–48.4
p-value*	0.0007	0.019	0.036	Not applicable
**MO-Y**				
Median	109.9	103.7	124.5	107.0
IQR	95.0–114	98.2–109.8	117.4–125.8	93.6–109.2
p-value*	0.45	0.89	0.011	Not applicable
**Automated IG count**				
Median	1.900	1.6	3.0	0.50
IQR	0.7–3.0	0.8–4.2	1.88–4.95	0.40–1.1
p-value*	0.006	0.04	0.012	Not applicable
**Abs auto IG (x10** ^**9**^ **/L)**				
Median	0.1400	0.19	0.26	0.05
IQR	0.10–0.44	0.08–0.64	0.15–0.56	0.03–0.11
p-value*	0.018	0.001	0.030	Not applicable

*The p-values are derived from comparison with the infection negative cases.

nCD64:lCD64, neutrophil CD64:lymphocyte CD64; mCD64:lCD64, monocyte CD64:lymphocyte CD64

nCD64:mHLA-DR, neutrophil CD64:monocyte HLA-DR; NE-WY, fluorescent light distribution width of the neutrophil area; NE-SFL, fluorescent light intensity of the neutrophil area; IG%, immature granulocyte percentage; CRP, C-reactive protein; Pct, procalcitonin; Abs auto IG, absolute automated IG count.

EDP testing was performed at a median of 4 hours following receipt of the FBC samples in the laboratory (IQR 2.3 to 8.5 hours). The biomarkers assessed were generally significantly higher among patients with bacteremia, non-bacteremic bacterial infection or TB as compared to those without infection. Exceptions to this included the NE-WY (which was not significantly increased in patients with either non-bacteremic bacterial infection or TB), the MO-Y (which was only significantly increased in patients with TB) and the mCD64:lCD64 (which was not significantly increased in any of the groups) (Table 1). Notably, the MO-Y was the only biomarker which was significantly higher among patients with TB as compared to those with bacteremia (p = 0.027). When comparing patients with TB and non-bacteremic bacterial infection, the nCD64:lCD64, n64:mHLA-DR and the MO-Y were all significantly higher among those with TB (p = 0.008, 0.007 and 0.025 respectively).

As the frequency of underlying HIV infection was substantially higher in the patients with infection (>50%) as compared to the infection negative group (28.6%), further analysis according to HIV-status was performed in order to ascertain whether the differences seen in the biomarkers assessed could be due to a confounding HIV-effect. This analysis revealed similar EDP results between the persons with and without HIV in each infection subgroup, although statistical significance on comparison with the infection negative group was largely lacking (possibly due to the limited sample size) (Table 2). Notably, three of the five individuals without HIV infection in the bacterial infection negative group were oncology patients undergoing routine surveillance for bacteremia. All had normal white cell counts and no laboratory evidence of infection. However, all three of these patients had a somewhat higher NE-SFL (median 48.2 (IQR 46.5–57.1)) than their other counterparts in the bacterial infection negative group (median = 44.7 (IQR 41.7–47.0)), and it is possible that this may have upwardly skewed the results in the bacterial infection negative group without HIV (thus obscuring significant differences due to infection).

**Table 2:** Biometrics and EDP data of samples with unequivocal results stratified according to HIV status.

Sample group	Bacteremic (BC positive) HIV positive	Bacteremic (BC positive) HIV negative	Non- bacteremic bacterial infection (BC negative, excluding TB), HIV positive	Non- bacteremic bacterial infection (BC negative, excluding TB), HIV neg	Bacterial infection negativeHIV pos	Bacterial infection negative HIV neg
**Number of samples**	8	6	9	7	2	5
**Male: Female**	0.14:1	0.2:1	0.8:1	0.8:1	0:1	0.25:1
**Median Age [IQR] (years)**	36 (25.8–53.8)	28 (13.4–29)	35.5 (25.5–47.8)	13 (2.4–37.3)	24 (21–27)	11 (1–47)
**Median CD4 count [IQR] (cells/ul)**	93 (58–406	Not applicable	145 (42–172)	Not applicable	Too few results for calculation	Not applicable
**nCD64:lCD64**						
Median	12.3	15.0	6.4	6.8	2.26	1.06
IQR	9.8–38.1	1.95–30.5	3.4–17.0	2.6–8.9	1.6–2.3	1–10.6
p-value*	0.003	0.048 (0.13ǂ)	0.01	0.027 (0.27ǂ)		
**mCD64:lCD64**						
Median	40.8	45.4	45.1	47.2	41.8	43.1
IQR	36.5–59.4	10.3–95.5	13.1–85.6	22.8–56.5	33.8–49.8	18.6–66.2
p-value*	0.43	0.97 (0.93ǂ)	0.95	0.69 (0.87ǂ)		
**nCD64:mHLA-DR**						
Median	2.42	0.75	0.84	0.54	0.33	0.07
IQR	0.92–9.1	0.61–1.2	0.2–3.1	0.13–0.82	0.04–0.62	0.02–0.43
p-value*	0.0003	0.0018 (0.017ǂ)	0.008	0.068 (0.149ǂ)		
**NE-WY**						
Median	806	837	793	755	655.0	788
IQR	710.8–943.3	766.5–1047.0	926–1384	660–893	617–693	702.5–846
p-value*	0.10	0.032 (0.25ǂ)	0.44	0.5 (0.93ǂ)		
**NE-SFL**						
Median	59.0	55.0	52.5	53.0	47.4	46.5
IQR	56.0–74.7	46.3–60.9	47.4–54.6	45.2–54.9	46.2–48.6	43.2–52.7
p-value*	0.0016	0.12 (0.33ǂ)	0.028	0.096 (0.33ǂ)		
**MO-Y**						
Median	106.1	107.2	104.4	100.9	115.8	103.8
IQR	95.5–116.3	94.8–115.1	101.6–119.3	95.3–107.3	(115.8–115.8)	93.6–108.4
p-value*	0.64	0.63 (0.54ǂ)	0.37	0.61 (1.0ǂ)		
**Automated IG count**						
Median	2.1	2.4	1.4	1.1	0.75	0.5
IQR	0.7–2.8	1.3–5.1	1.1–6.9	0.6–3.1	0.7–0.8	0.5–1.7
p-value*	0.07	0.0065 (0.035ǂ)	0.025	0.13 (0.34ǂ)		
**Abs auto IG (x10** ^**9**^ **/L)**						
Median	0.14	0.29	0.16	0.19	0.09	0.04
IQR	0.04–0.38	0.13–0.8	0.1–0.86	0.06–0.45	0.05–0.12	0.03–0.25
p-value*	0.20	0.0085 (0.052ǂ)	0.032	0.032 (0.06ǂ)		

*The p-values are derived from comparison with the infection negative cases.

ǂ P-vales were derived from comparison to the bacterial infection negative group without HIV. Unfortunately, the number of bacterial infection negative cases in people with HIV was too small to allow for this comparison in the patients with HIV.

nCD64:lCD64, neutrophil CD64:lymphocyte CD64; mCD64:lCD64, monocyte CD64:lymphocyte CD64

nCD64:mHLA-DR, neutrophil CD64:monocyte HLA-DR; NE-WY, fluorescent light distribution width of the neutrophil area; NE-SFL, fluorescent light intensity of the neutrophil area; IG%, immature granulocyte percentage; CRP, C-reactive protein; Pct, procalcitonin; Abs auto IG, absolute automated IG count.

### Receiver operating characteristic (ROC) curve analysis

For the purposes of ROC curve analysis, patients with TB were included among the non-bacteremic bacterial infection cases owing to the small sample size of this group.

The best performance for distinguishing patients with bacteremia from those without infection was seen with the nCD64:mHLA-DR at a cut-off value of 0.34 (Table 3). Among the automated parameters, the NE-SFL showed the best performance (AUC 0.84) (Table 3) at a cut-off value of 49.75. The CRP and PCT also demonstrated excellent performance, but were only measured in a proportion of the patients (Table 2). None of the parameters demonstrated good performance for distinguishing patients with bacteremia from those with non-bacteremic bacterial infection (AUC <0.8) (S2 Table). On stratification of the analysis according to HIV status, the NE-SFL was found to be a significant predictor of bacteremia among the persons with HIV infection at a cut-off value of 49.75 (AUC = 1), but not so among the individuals without HIV (S3 and S4 Tables). In contrast, the IG% was a significant predictor of bacteremia in the HIV negative group, but not among the HIV positive patients (S3 and S4 Tables). Notably, the NE-SFL also showed a very high statistically significant negative predictive value for discriminating between bacteremic and non-bacteremic bacterial infection among the persons with HIV at a cut-off value of 55.2 (S5 Table), while none of the parameters were helpful in this regard in the group without HIV infection (S6 Table).

**Table 3:** ROC curve analysis assessing the various biomarkers among patients with bacteremic infection compared to those with no evidence of bacterial infection.

Parameter	AUC	95% CI	p-value for AUC	LR	Sensitivity	Specificity	Cut off value	NPV	PPV
					(%)	(%)		(%)	(%)
**nCD64: lCD64**	0.86	0.75–0.98	0.0003	4.25	92.3	78.3	> 7.4	70.6	94.7
**nCD64: mHLA-DR**	0.95	0.88–1.02	< 0.0001	19.5	84.6	95.7	> 0.34	90.9	88.0
**NE-WY**	0.78	0.63–0.93	0.005	3.54	61.5	82.6	> 750.5	66.7	79.2
**NE-SFL**	0.84	0.70–0.98	0.0007	4.87	84.6	82.6	> 49.75	73.3	90.5
**Automated IG%**	0.78	0.62–0.93	0.006	2.65	69.2	73.9	> 0.85	60.0	81.0
**Abs auto IG**	0.74	0.57–0.91	0.017	3.98	69.2	82.6	> 0.065	69.2	82.6
**CRP** ^**+**^	1	1.00–1.00	0.0002	>17	100	100	> 12.5	100.00	95.0
**PCT** *	1	1.00–1.00	0.037	>8	100	100	>0.48	100.0	100.0

^+^ N = 24

*N = 10

AUC, area under the curve; CI, confidence interval; LR, likelihood ratio; NPV, negative predictive value; PPV, positive predictive value; nCD64:lCD64, neutrophil CD64:lymphocyte CD64; nCD64:mHLA-DR, neutrophil CD64:monocyte HLA-DR; NE-WY, fluorescent light distribution width of the neutrophil area; NE-SFL, fluorescent light intensity of the neutrophil area; IG%, immature granulocyte percentage; CRP, C-reactive protein; Abs auto IG, absolute automated IG count.

### Cohen’s Kappa co-efficient

Agreement between the EDPs and the ratio of nCD64:mHLA-DR expression (as the sepsis biomarker performed in all patients with the best overall performance) was determined by Cohen’s kappa co-efficient on all the tested samples (N = 151) using the cut-off values determined by ROC analysis above. This revealed moderate agreement between the NE-SFL and the nCD64:mHLA-DR (kappa = 0.5). Fair agreement was noted with the automated IG count (kappa = 0.38) and NE-WY (kappa = 0.3). Notably, the CRP showed only slight agreement with the nCD64:mHLA-DR (kappa = 0.18).

### Analysis of the correlation between the NE-SFL and nCD64 and the MO-Y and mCD64 expression, respectively

**Fig 1:**
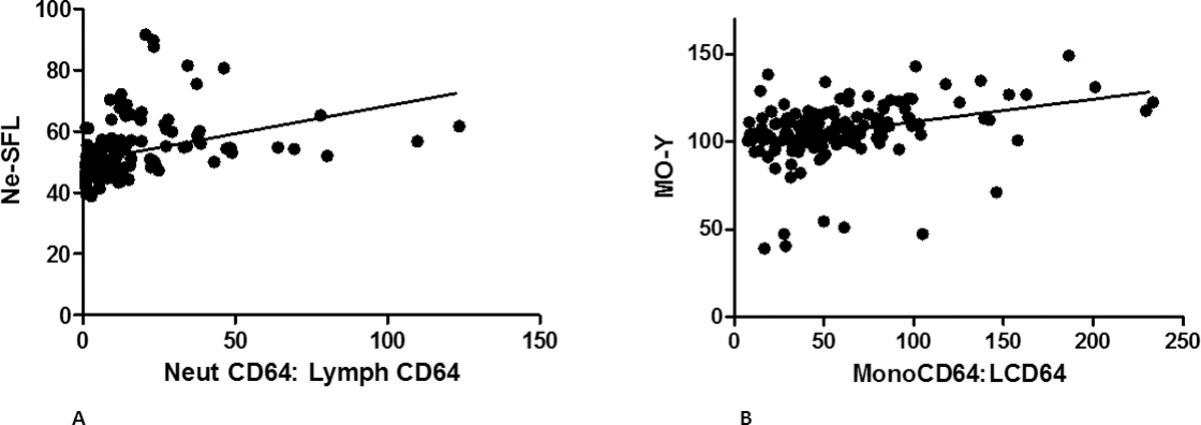
Graphs depicting the linear regression analysis between NE-SFL and nCD64:lCD64 (A) and between MO-Y and mCD64:lCD64 (B).

In order to assess whether the EDPs NE-SFL and MO-Y correlated with neutrophil and monocyte activation respectively, we assessed the expression of CD64 on monocytes and neutrophils flow cytometrically and calculated ratios of nCD64:lCD64 and mCD64:lCD64, respectively. We then performed linear regression analysis and found weak positive correlations (r2 = 0.14, p<0,0001 for the NE-SFL and r2 = 0.10, p<0,0001 for the MO-Y) (Fig 1), suggesting that there is an association between these parameters and neutrophil and monocyte activation, respectively.

### Analysis of the agreement between the automated and manual IG counts

A previous South African study showed that the automated IG count was inaccurate in a proportion of patients, particularly in those with underlying HIV infection [20]. On comparison of the manual and automated differential counts in this study population, 85 samples had an automated IG count ≥1% and of these, 8 (9.4%) were grossly discordant with the manual IG count (median manual count 1.25% vs median automated count 12.4%). HIV-status was documented in 6 of these, with a seropositivity rate of 83.3%. Among all cases with HIV infection, 5 of the 43 samples (11.6%) had gross erroneous elevation of their IG counts.

## Discussion

This study assessed EDPs for the detection of bacteremic infection in a South African laboratory with a high HIV-seropositivity rate. All EDPs evaluated are performed concurrently with the differential white cell count on Sysmex haematology analysers, and therefore hold appeal in the resource constrained setting. The NE-SFL performed well when comparing bacteremic against bacterial infection negative samples, with a sensitivity and specificity of 84.6% and 82.6% respectively at a cut off values of >49.75. These results are similar to those previously reported by Park *et al*., who showed the NE-SFL to have sensitivity and specificity of 71.3% and 86.2% at a cut off value of >51.6 in a study comparing samples from patients with sepsis to normal controls [11]. Furthermore, a significant correlation was found between the NE-SFL and the ratio of nCD64:lCD64 expression, which suggests that an elevated NE-SFL does reflect neutrophil activation as hypothesized by Park *et al*. [11] Interestingly, the performance of the NE-SFL was substantially better among the patients with HIV infection for discriminating those with bacteremia from those without bacterial infection (AUC = 1), and also showed a strong negative predictive value (91.7%) for the discrimination between bacteremic and non-bacteremic bacterial infection at a cut-off value of 55.2 (AUC = 0.79). The reason for the superior performance according to HIV status is unclear, but may relate to differences in the severity of sepsis (and consequent neutrophil activation) among immunocompromised patients with bateremia. Alternatively, the poorer performance in the HIV negative group may be due to the relatively large proportion of patients in the bacterial infection negative group without HIV who were oncology patients undergoing routine surveillance for bacteremia. Although these individuals all had normal white cell counts and no laboratory evidence of infection, they had higher NE-SFL levels than their other infection negative counterparts, possibly due to an unknown effect on the neutrophils due to recent chemotherapy exposure. It is possible that this may have masked a significant difference in NE-SFL levels due to infection in the HIV negative cohort. Further study in this regard (including detailed clinical information) would be of interest. The NE-SFL was also significantly elevated amongst patients with TB, as was the MO-Y (which is thought to reflect monocyte activation). Notably, the MO-Y was increased only among patients with TB, and near normal among patients with bacterial infection (both bacteremic and non-bacteremic). TB is quite often difficult to diagnose, even where molecular techniques are utilised. The possibility of a sensitive and specific EDP biomarker is consequently of interest, and further studies on a larger number of patients with TB would be of value.

In contrast to the NE-SFL (which is currently not universally available to all Sysmex users), the automated IG count is reported as part of the six-part differential count on XT-, XE- and XN-series Sysmex analysers. This parameter was first reported to be of utility in detecting bacterial infection by Ansari-Lari *et al*., with a specificity of >90% for detecting bacteremia among samples submitted for a blood culture at an IG count >3% [5]. The sensitivity of this parameter was however low in this study (35–40%). It has since been shown to have utility in either diagnosing or stratifying sepsis severity in a number of clinical contexts, including in the ICU [5, 13–15], the emergency department [16, 17], burn victims [18] and patients admitted in hematology and infectious diseases departments [29]. The ideal cut-off value for interpretation of IG results is not well standardised, with different studies reporting superior performance with counts >0.5% [11, 16], >2% [19], >3% [5] or with elevation of the absolute IG count [14, 17, 18]. As compared to the findings observed by Ansari-Lari *et al*., in our study this parameter showed only modest performance in discriminating bacteremia from bacterial infection negative cases (AUC = 0.78, with sensitivity of 69.2% and specificity of 73.9% at a cut-off value of 0.85%). However, on analysis according to HIV status, the IG% was shown to be the best performing EDP in differentiating bacteremia from bacterial infection negative cases among people without HIV infection, with a sensitivity of 80% and specificity of 83.3% at a cut-off of 1.35% (AUC = 0.9). In contrast, the performance of the automated IG% among the patients with HIV infection was poor (AUC = 0.69), likely due to the spurious elevation of the IG% count in 11.6% of the patients with HIV. The latter finding is in agreement with those of a previous South African study [20], and confirm that the IG% is not a valuable sepsis biomarker in the setting of HIV infection.

Results of nCD64 analysis among the bacteremic samples are consistent with those published in a meta-analysis by Yeh *et al*., showing that nCD64 expression is a good biomarker of sepsis. In this study population, the nCD64:mHLA-DR out-performed the nCD64:lCD64, with a sensitivity, specificity and AUC of 84.6%, 95.7% and 0.95 (95% CI 0.88–1.02), respectively. Agreement between the EDPs and the nCD64:mHLA-DR ratio (as the sepsis biomarker measured in all the samples which was demonstrated to have the best overall performance) revealed moderate agreement for the NE-SFL. Given the high cost and specialised nature of flow cytometry-based tests, this finding further supports using this parameter over the costly nCD64:mHLADR in resource limited settings.

The CRP showed outstanding performance for the detection of bacteremia in this study when using a cut-off value of >12.50. This excellent performance is likely owing to the study design, which pre-selected a population that is highly likely to have infection. This lowers the possibility of false elevation of the CRP. Interestingly, there was very poor agreement between the CRP and the nCD64:mHLA-DR, which may reflect the non-specific nature of the CRP, being an acute phase reactant elevated in many other inflammatory conditions [30]. In contrast, the nCD64:mHLA-DR could be more specific to the immune response to infection [31]. Notably, the weaknesses in this study’s design which are likely to have skewed the CRP result also apply to the EDPs, and further assessment in a broader sample (without pre-selection for clinical evidence of infection) is needed.

### Limitations

This study has several limitations. As discussed above, the study design has preselected for patients likely to have infection, and assessment of the performance of these parameters in a broader patient cohort is required. In addition, we have not included a completely healthy control group for definitive comparison of pathological versus non-pathological samples. While this does reflect routine hospital practise (where the identification of infection among ill people is needed), it also creates some ambiguity in the findings. Furthermore, this study has included a very heterogeneous patient population, and the impact of factors known to cause false positives in other sepsis biomarkers (such as auto-immune disease and cancer) is therefore obscured. Lastly, the study is somewhat under-powered for the HIV sub-analysis which was performed, and the findings require confirmation in a larger sample. This is particularly the case in the patients with TB, as this subgroup included only four cases.

## Conclusion

EDPs offer great potential as a marker of sepsis in that they are cost-efficient and readily available. In this study, the NE-SFL showed the greatest promise as a biomarker of bacteremia among patients with HIV, while the automated IG% showed the best performance among those without HIV. In addition, the MO-Y showed potential as a possible specific biomarker for TB. Further studies on the value of the NE-SFL and the IG% in unselected patients, as well as assessment of the MO-Y in a larger number of patients with TB would be of value.

## Supporting information

S1 Table Median percentage changes in results for each of the EPD parameters at increasing time intervals when stored at room temperature and when refrigerated at 2–8 deg celsius. (DOCX)

S2 Table ROC curve analysis assessing the various biomarkers among patients with bacteremic infection compared to those with non-bacteraemic bacterial infection. (DOCX)

S3 Table ROC curve analysis assessing the various biomarkers among persons with HIV infection with bacteremic infection compared to those without bacterial infection. (DOCX)

S4 Table ROC curve analysis assessing the various biomarkers among HIV negative patients with bacteremic infection compared to those without bacterial infection. (DOCX)

S5 Table ROC curve analysis assessing the various biomarkers among persons with HIV with bacteremic infection compared to those with non-bacteraemic bacterial infection. (DOCX)

S6 Table ROC curve analysis assessing the various biomarkers among HIV negative patients with bacteremic infection compared to those with non-bacteremic bacterial infection. (DOCX)

S1 Dataset Minimal dataset. (XLSX)

S1 Appendix Supplementary methods. (PDF)
