# Supplementary material for: The utility of extended differential parameters as a biomarker of bacteremia at a tertiary academic hospital in persons with and without HIV infection in South Africa
Source: PLoS One. 2022 Feb 17;17(2):e0262938. doi: 10.1371/journal.pone.0262938 (PMC8853519; doi:10.1371/journal.pone.0262938)
Supplement: S1 Table — (DOCX) [file pone.0262938.s001.docx]

**S1 Table**: Median percentage changes in results for each of the EPD parameters at increasing time intervals when stored at room temperature and when refrigerated at 2-8 deg Celsius

| **Time interval** | **NE-SFL**  **2-8 deg C** | **NE-SFL**  **room temperature** | **MO-Y**  **2-8 deg C** | **MO-Y**  **room temperature** | **NE-WY**  **2-8 deg C** | **NE-WY**  **room temperature** |
| --- | --- | --- | --- | --- | --- | --- |
| 6 hrs (%) | 3,8 | 0,4 | -4,0 | -1,4 | -3,7 | -0,3 |
| 12hrs (%) | 3,7 | -1,6 | -1,0 | -3,5 | -1,1 | 4,9 |
| 18 hrs (%) | 6,6 | -1,3 | -3,0 | -2,7 | 1,4 | 7,3 |
| 24 hrs (%) | -0,7 | -1,3 | -1,6 | -2,2 | 4,4 | 8,4 |
| 36hrs (%) | 2,9 | -1,3 | 0,1 | -5,9 | 5,0 | 14,7 |
| 48 hrs (%)* | 3,6 |  | -2,4 |  | 7,2 |  |
| 72 hrs (%) | -1,4 | -3,7 | -8,2 | -26,2 | 15,6 | 38,7 |

*Analysis on the samples stores at room temperature was erroneously omitted at 48 hours.
