# Supplementary material for: The utility of extended differential parameters as a biomarker of bacteremia at a tertiary academic hospital in persons with and without HIV infection in South Africa
Source: PLoS One. 2022 Feb 17;17(2):e0262938. doi: 10.1371/journal.pone.0262938 (PMC8853519; doi:10.1371/journal.pone.0262938)
Supplement: S2 Table — (DOCX) [file pone.0262938.s002.docx]

**S2 Table.** ROC curve analysis assessing the various biomarkers among patients with bacteremic infection compared to those with non-bacteraemic bacterial infection.

| **Parameter** | **AUC** | **95% CI** | **p-value for AUC** | **LR** | **Sensitivity**  **(%)** | **Specificity**  **(%)** | **Cut off value** | **NPV**  **(%)** | **PPV**  **(%)** |
| --- | --- | --- | --- | --- | --- | --- | --- | --- | --- |
| **nCD64: lCD64** | 0.77 | 0.63 -0.91 | 0.0006 | 4.05 | 75 | 81.5 | > 9.1 | 80.8 | 62.0 |
| **nCD64: mHLA-DR** | 0.76 | 0.64 – 0.89 | 0.0007 | 5.79 | 64.3 | 88.9 | > 0.61 | 85.7 | 58.8 |
| **NE-WY** | 0.65 | 0.51 - 0.80 | 0.055 | 2.70 | 46.9 | 78.3 | > 757 | 78.9 | 52.8 |
| **NE-SFL** | 0.74 | 0.61 - 0.80 | 0.002 | 2.95 | 79.3 | 73.1 | > 54.5 | 85.7 | 72.0 |
| **Automated IG%** | 0.52 | 0.36 - 0.68 | 0.78 | 1.80 | 31.3 | 82.6 | > 3.6 | 53.7 | 28.6 |
| **Abs auto IG** | 0.58 | 0.43 - 0.74 | 0.31 | 1.44 | 68.8 | 52.2 | > 0.15 | 45.5 | 33.3 |

AUC, area under the curve; CI, confidence interval; LR, likelihood ratio; NPV, negative predictive value; PPV, positive predictive value; nCD64:lCD64, neutrophil CD64:lymphocyte CD64; nCD64:mHLA-DR, neutrophil CD64:monocyte HLA-DR; NE-WY, fluorescent light distribution width of the neutrophil area; NE-SFL, fluorescent light intensity of the neutrophil area; IG%, immature granulocyte percentage; Abs auto IG, absolute automated IG count.
