# Supplementary material for: The utility of extended differential parameters as a biomarker of bacteremia at a tertiary academic hospital in persons with and without HIV infection in South Africa
Source: PLoS One. 2022 Feb 17;17(2):e0262938. doi: 10.1371/journal.pone.0262938 (PMC8853519; doi:10.1371/journal.pone.0262938)
Supplement: S3 Table — (DOCX) [file pone.0262938.s003.docx]

**S3 Table.** ROC curve analysis assessing the various biomarkers among persons with HIV infection with bacteremic infection compared to those without bacterial infection.

| **Parameter** | **AUC** | **95% CI** | **p-value for AUC** | **LR** | **Sensitivity**  **(%)** | **Specificity**  **(%)** | **Cut off value** | **NPV (%)** | **PPV (%)** |
| --- | --- | --- | --- | --- | --- | --- | --- | --- | --- |
| **nCD64: lCD64** | 0.87 | 0.65 - 1.1 | 0.12 | 8.0 | 100 | 87.5 | >6.2 | 66.7 | 100 |
| **nCD64: mHLA-DR** | 0.93 | 0.71 – 1.1 | 0.07 | 8.0 | 100 | 87.5 | > 0.68 | 66.7 | 100 |
| **NE-WY** | 0.87 | 0.62 – 1.1 | 0.12 | 4.0 | 100 | 75 | > 745 | 100 | 50 |
| **NE-SFL** | 1 | 1 - 1 | 0.037 | >8 | 100 | 100 | > 49.75 | 100 | 100 |
| **Automated IG%** | 0.69 | 0.36- 1.01 | 0.4 | 2.67 | 100 | 62.5 | > 1.35 | 40 | 100 |
| **Abs auto IG** | 0.63 | 0.27 - 0.98 | 0.62 | 2.0 | 100 | 50 | > 0.14 | 33.3 | 100 |

AUC, area under the curve; CI, confidence interval; LR, likelihood ratio; NPV, negative predictive value; PPV, positive predictive value; nCD64:lCD64, neutrophil CD64:lymphocyte CD64; nCD64:mHLA-DR, neutrophil CD64:monocyte HLA-DR; NE-WY, fluorescent light distribution width of the neutrophil area; NE-SFL, fluorescent light intensity of the neutrophil area; IG%, immature granulocyte percentage; Abs auto IG, absolute automated IG count.
