# Supplementary material for: The utility of extended differential parameters as a biomarker of bacteremia at a tertiary academic hospital in persons with and without HIV infection in South Africa
Source: PLoS One. 2022 Feb 17;17(2):e0262938. doi: 10.1371/journal.pone.0262938 (PMC8853519; doi:10.1371/journal.pone.0262938)
Supplement: S4 Table — (DOCX) [file pone.0262938.s004.docx]

**S4 Table.** ROC curve analysis assessing the various biomarkers among HIV negative patients with bacteremic infection compared to those without bacterial infection.

| **Parameter** | **AUC** | **95% CI** | **p-value for AUC** | **LR** | **Sensitivity**  **(%)** | **Specificity**  **(%)** | **Cut off value** | **NPV (%)** | **PPV (%)** |
| --- | --- | --- | --- | --- | --- | --- | --- | --- | --- |
| **nCD64: lCD64** | 0.80 | 0.52 - 1.1 | 0.10 | >3.6 | 60 | 100 | >1.45 | 100.0 | 75.0 |
| **nCD64: mHLA-DR** | 0.93 | 0.78 – 1.1 | 0.018 | >4.8 | 80 | 100 | > 0.35 | 100.0 | 85.7 |
| **NE-WY** | 0.83 | 0.57 – 1.1 | 0.088 | >3.0 | 50 | 100 | > 750.5 | 100.0 | 66.7 |
| **NE-SFL** | 0.7 | 0.37 – 1.0 | 0.27 | 3.6 | 60 | 83.3 | > 47.3 | 75.0 | 71.4 |
| **Automated IG%** | 0.9 | 0.7 - 1.09 | 0.029 | 4.8 | 80 | 83.3 | > 1.35 | 80.0 | 83.3 |
| **Abs auto IG** | 0.87 | 0.61- 1.10 | 0.045 | >4.8 | 80 | 100 | > 0.085 | 100.0 | 85.7 |

AUC, area under the curve; CI, confidence interval; LR, likelihood ratio; NPV, negative predictive value; PPV, positive predictive value; nCD64:lCD64, neutrophil CD64:lymphocyte CD64; nCD64:mHLA-DR, neutrophil CD64:monocyte HLA-DR; NE-WY, fluorescent light distribution width of the neutrophil area; NE-SFL, fluorescent light intensity of the neutrophil area; IG%, immature granulocyte percentage; Abs auto IG, absolute automated IG count.
