# Supplementary material for: The utility of extended differential parameters as a biomarker of bacteremia at a tertiary academic hospital in persons with and without HIV infection in South Africa
Source: PLoS One. 2022 Feb 17;17(2):e0262938. doi: 10.1371/journal.pone.0262938 (PMC8853519; doi:10.1371/journal.pone.0262938)
Supplement: S5 Table — (DOCX) [file pone.0262938.s005.docx]

**S5 Table.** ROC curve analysis assessing the various biomarkers among persons with HIV with bacteremic infection compared to those with non-bacteraemic bacterial infection.

| **Parameter** | **AUC** | **95% CI** | **p-value for AUC** | **LR** | **Sensitivity**  **(%)** | **Specificity**  **(%)** | **Cut off value** | **NPV (%)** | **PPV (%)** |
| --- | --- | --- | --- | --- | --- | --- | --- | --- | --- |
| **nCD64: lCD64** | 0.78 | 0.57 – 0.99 | 0.03 | 8 | 66.2 | 91.7 | >8.22 | 87.5 | 50.0 |
| **nCD64: mHLA-DR** | 0.63 | 0.38 – 0.89 | 0.31 | 2.15 | 53.9 | 75 | > 1.35 | 80.0 | 50.0 |
| **NE-WY** | 0.57 | 0.31 – 0.83 | 0.61 | 1.85 | 46.2 | 75 | > 795 | 75.0 | 42.9 |
| **NE-SFL** | 0.79 | 0.58 – 1.0 | 0.03 | 6.2 | 76.9 | 87.5 | > 55.2 | 91.7 | 70.0 |
| **Automated IG%** | 0.59 | 0.33 - 0.85 | 0.49 | 3.08 | 38.5 | 87.5 | > 3.45 | 53.3 | 14.3 |
| **Abs auto IG** | 0.66 | 0.41- 0.91 | 0.23 | 1.69 | 84.6 | 50 | > 0.12 | 42.9 | 26.7 |

AUC, area under the curve; CI, confidence interval; LR, likelihood ratio; NPV, negative predictive value; PPV, positive predictive value; nCD64:lCD64, neutrophil CD64:lymphocyte CD64; nCD64:mHLA-DR, neutrophil CD64:monocyte HLA-DR; NE-WY, fluorescent light distribution width of the neutrophil area; NE-SFL, fluorescent light intensity of the neutrophil area; IG%, immature granulocyte percentage; Abs auto IG, absolute automated IG count.
