# Supplementary material for: The utility of extended differential parameters as a biomarker of bacteremia at a tertiary academic hospital in persons with and without HIV infection in South Africa
Source: PLoS One. 2022 Feb 17;17(2):e0262938. doi: 10.1371/journal.pone.0262938 (PMC8853519; doi:10.1371/journal.pone.0262938)
Supplement: S6 Table — (DOCX) [file pone.0262938.s006.docx]

**S6 Table.** ROC curve analysis assessing the various biomarkers among HIV negative patients with bacteremic infection compared to those with non-bacteremic bacterial infection.

| **Parameter** | **AUC** | **95% CI** | **p-value for AUC** | **LR** | **Sensitivity**  **(%)** | **Specificity**  **(%)** | **Cut off value** | **NPV (%)** | **PPV (%)** |
| --- | --- | --- | --- | --- | --- | --- | --- | --- | --- |
| **nCD64: lCD64** | 0.5 | 0.10 – 0.90 | 1 | 2 | 100 | 50 | >2.23 | 25.0 | 30.0 |
| **nCD64: mHLA-DR** | 0.7 | 0.40 – 1.02 | 0.20 | 4.3 | 71.4 | 83.3 | > 0.6 | 83.3 | 71.4 |
| **NE-WY** | 0.69 | 0.38 – 1.0 | 0.25 | >3.43 | 57.1 | 57.1 | > 757 | 100.0 | 66.7 |
| **NE-SFL** | 0.64 | 0.31 – 0.98 | 0.39 | 2.1 | 71.4 | 66.7 | > 53.9 | 71.4 | 66.7 |
| **Automated IG%** | 0.69 | 0.39 - 0.99 | 0.25 | 3.43 | 57.1 | 83.3 | > 1.25 | 80.0 | 62.5 |
| **Abs auto IG** | 0.62 | 0.30- 0.94 | 0.48 | >2.57 | 42.9 | 100 | > 0.095 | 100.0 | 60.0 |

AUC, area under the curve; CI, confidence interval; LR, likelihood ratio; NPV, negative predictive value; PPV, positive predictive value; nCD64:lCD64, neutrophil CD64:lymphocyte CD64; nCD64:mHLA-DR, neutrophil CD64:monocyte HLA-DR; NE-WY, fluorescent light distribution width of the neutrophil area; NE-SFL, fluorescent light intensity of the neutrophil area; IG%, immature granulocyte percentage; Abs auto IG, absolute automated IG count.
