# Supplementary material for: The utility of extended differential parameters as a biomarker of bacteremia at a tertiary academic hospital in persons with and without HIV infection in South Africa
Source: PLoS One. 2022 Feb 17;17(2):e0262938. doi: 10.1371/journal.pone.0262938 (PMC8853519; doi:10.1371/journal.pone.0262938)
Supplement: S1 Appendix — (PDF) [file pone.0262938.s008.pdf]

Supplementary methods

Neutrophil CD64 expression was measured on the sample submitted for a FBC by flow cytometry using an adaptation of a previously described protocol [9] on a Beckman Coulter Navios flow cytometer (Beckman Coulter, Inc, Brea, California, USA). Daily quality control of the flow cytometer includes monitoring the background count, carryover between samples, analysis of Flow Check^{PRO} to confirm optical alignment and fluidics and Flow Set ^{PRO} for standardizing the forward scatter, side scatter and fluorescence channels. Beckman Coulter Immunotrol process controls were used daily on the automated Beckman Coulter T-Q-Prep^{TM} instruments to verify the lyse, stabilize and fixing procedures. Analysis was performed within 36 hours after blood sampling to ensure viability of cellular components. A lyse-no-wash preparation method using commercially produced fluorescence conjugated antibodies with specificity to white blood cells (CD45), monocytes (CD14), HLA-DR and CD64 and a fluorescence latex bead suspension was used. Briefly, 100 µL of whole blood was incubated for 15 minutes in the dark at room temperature with 10µl of Beckman HLA-DR (FITC), CD64 PE and CD45 ECD monoclonal antibodies followed by red cell lysis using the Beckman Coulter T-Q-Prep/ Immunoprep^{TM} reagent system. Beckman Coulter Flow Count^{TM} beads (100µL) were then added (to monitor stability of the mean fluorescent intensity with each sample) and flow cytometer acquisition and analysis performed on a minimum of 50 000 leukocytes. Data analysis for fluorescence intensity was performed on the Beckman Navios flow cytometer. Live gating was performed according to light scatter for different populations (S1-2 Figs). CD64 and HLA-DR mean fluorescence intensity (MFI) was measured as a linearized value of log scale on the lymphocytes (negative control), the monocytes (positive control), the neutrophils, and the Flow Count^{TM} beads.

The MFI for the Flow Count™ beads remained stable throughout the study, with a mean of 642.6 (cv 5.08%) hence the values for MFI for patient samples could be taken directly from the printout.

The MFI for CD64 and HLA-DR was recorded for neutrophils, lymphocytes and monocytes. The ratio of CD64 expression for neutrophils to lymphocytes as well as the ratio for neutrophil CD64 expression to monocyte HLA-DR expression were then calculated.

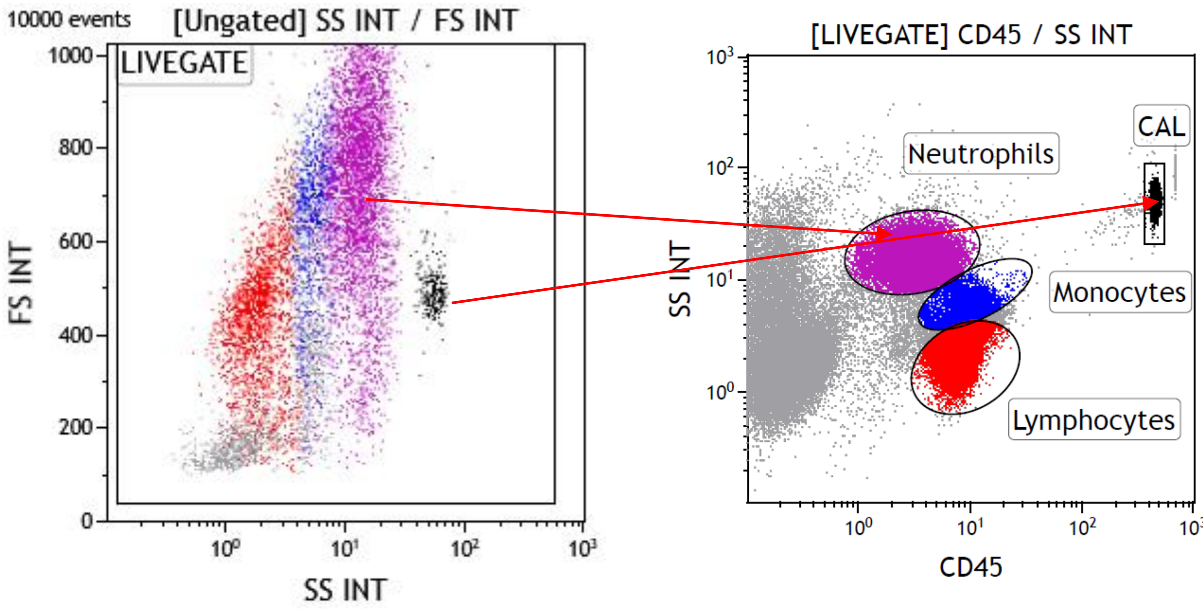

(A) (B)

S1 Fig. Live gating strategy for identifying populations of interest. The granulocytes (purple events), monocytes (blue events), lymphocytes (red events) and Flow Count™ beads (black events) are highlighted and gated as side versus forward scatter (A) and as CD45 versus side scatter (B).

A

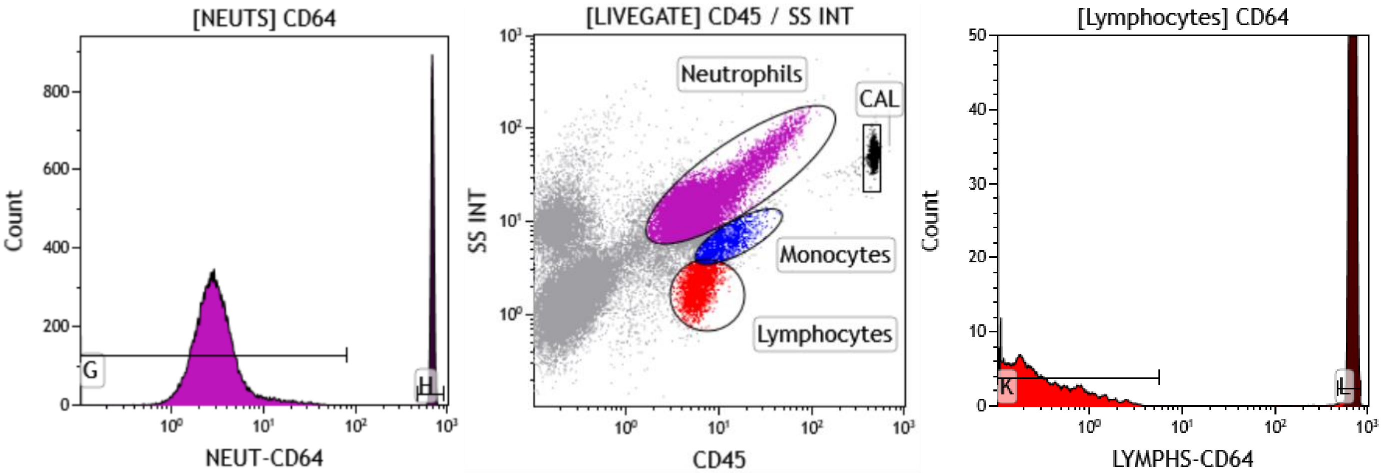

Gate	X-Med
All	3,25
G	2,86
H	684,18

Gate	X-Med
All	0.10
K	0.10
L	686.72

(a) (b) (c)

B

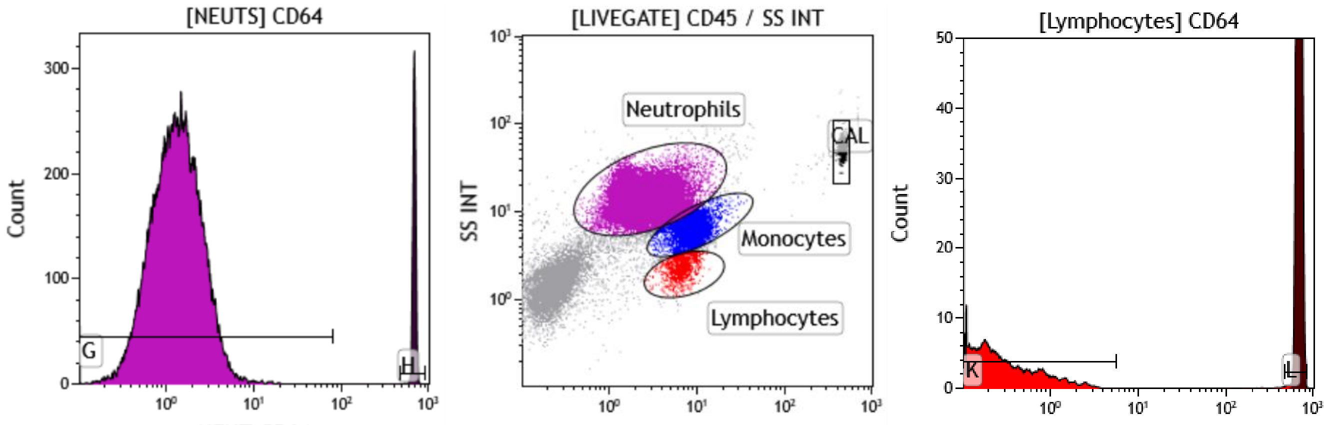

(a) (b) (c)

C

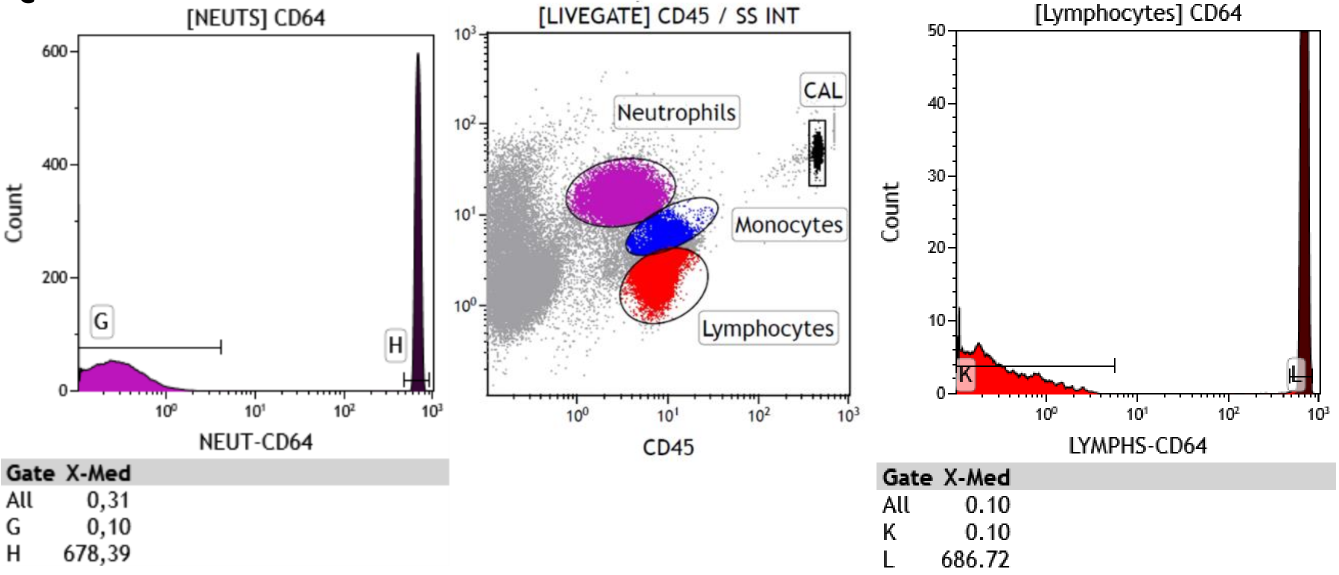

(a) (b) (c)

S2 Fig. Flow cytometry live gating strategy to identify populations of interest, namely granulocytes, monocytes and lymphocytes (b). Each gated population feeds into CD64 expression histograms for neutrophils (a) and lymphocytes (c), from where the ratio of CD64 expression was calculated.

A. Shows a bacteremic sample - The MFI is read off the histogram plots, such that neutrophil CD64 expression is 2.86 (G) and lymphocyte CD64 expression is 0.10 (K).

The beads remain constant in both the histograms (H and L respectively). The nCD64:lCD64 ratio was calculated as 28.6:1 in this case.

B. Shows a sample with non-bacteremic bacterial infection - The MFI is read off the histogram plots, such that neutrophil CD64 expression is 1.31 (G) and lymphocyte CD64 expression is 0.10 (K). The beads remain constant in both the histograms (H and L respectively). The nCD64:lCD64 ratio was calculated as 13.1:1 in this case.

C. Shows a samples with no bacterial infection - The MFI is read off the histogram plots, such that neutrophil CD64 expression is 0.10 (G) and lymphocyte CD64 expression is 0.10 (K). The beads remain constant in both the histograms (H and L respectively). The nCD64:lCD64 ratio was calculated as 1:1 in this case.
